# Endoscopic combined intrarenal surgery for successful removal of an encrusted ureteral stent and multiple renal stones in solitary kidney: A case report

**DOI:** 10.1016/j.ijscr.2024.109324

**Published:** 2024-02-01

**Authors:** Yufi Aulia Azmi, Johan Renaldo, Dimas Panca Andhika

**Affiliations:** a Department of Urology, Faculty of Medicine Universitas Airlangga – Dr. Soetomo General Academic Hospital, Surabaya, Indonesia; b Department of Urology, Faculty of Medicine Universitas Airlangga – Universitas Airlangga Academic Hospital, Surabaya, Indonesia; c Department of Health Sciences, University of Groningen, University Medical Center Groningen, Groningen, the Netherlands

**Keywords:** Neglected DJ stent, Infection, Endoscopic intrarenal surgery, Recurrent multiple kidney stones, Solitary kidney, Case reports

## Abstract

**Introduction and importance:**

Several endoscopic procedures have been performed to clear stones in the kidney. A potential technique called endoscopic combined intrarenal surgery (ECIRS) is a practical option for treating complex kidney stones. Hereby, we report a neglected double j (DJ) stent and recurrent multiple kidney stones treated by ECIRS.

**Case presentation:**

A 56-year-old female complained of right flank pain one week ago, which worsened within one day before hospital admission. She underwent DJ stent insertion one year ago because of residual stones after right percutaneous nephrolitothomy (PCNL) but was lost to follow-up. There was a history of left nephrectomy, leaving the patient with only a right kidney. A x-ray kidney ureter bladder (KUB) evaluation showed multiple irregular radiopaque shadows. A computed tomography (CT) scan detected numerous stones in the right kidney. The patient underwent ECIRS to remove the DJ stent and clear multiple stones in the right kidney. Following the surgery, the patient was discharged from the hospital on the fourth postoperative day with no complications and residual stone.

**Clinical discussions:**

ECIRS is a technique that combines a simultaneous antero-retrograde approach to the kidney and aims to resolve nephrolithiasis in one step and with one access. The ECIRS procedure could be considered in cases of complex multiple stone nephrolithiasis. ECIRS could widen the operative vision, thus helping to clear difficult kidney stones.

**Conclusions:**

The ECIRS technique could provide successful and safe management of recurrent multiple kidney stones and encrusted, neglected DJ stents in a solitary kidney patient.

## Highlights

• Endoscopic Combined Intra Renal Surgery (ECIRS) is a practical option for treating complex kidney stones.
• ECIRS is a technique that combines a simultaneous Antero-retrograde approach to the kidney
• ECIRS achieves high stone-free rates with low complication rates
• The ECIRS technique could provide successful and safe management

## 1 Introduction

Nephrolithiasis is a condition of the presence of stones or abnormal sediment in the kidneys. It could cause obstruction in the flow of urine and result in damage to the kidney nephrons [1]. There are currently several treatment options for nephrolithiasis, such as open nephrolithotomy, extracorporeal shock wave lithotripsy (ESWL), flexible ureteroscopy (fURS), PCNL, and laparoscopy [2]. However, in some cases it is difficult to remove all stones from each renal medulla using a single surgical technique and equipment. A potential technique called ECIRS, is a practical option for treating complex kidney stones [2]. Prior research has indicated that ECIRS is a safe and efficient therapeutic intervention, particularly for patients with large and intricate nephrolithiasis [3]. When the DJ stent remains in the urinary tract for too prolonged a period of time, minerals in the urine accumulate on the surface of the DJ stent and cause encrustation [4]. This procedure has demonstrated a significantly high one-step stone-free rate (SFR), a diminished need for supplementary procedures, and a low incidence of complications [3]. In addition, using a flexible endoscope during ECIRS minimizes radiation exposure, bleeding risk, and post-PCNL kidney damage [5]. Thus, our study exhibits the efficacious implementation of ECIRS in a solitary kidney afflicted with neglected DJ stent repeated occurrences of multiple kidney stones, which are classified as intricate cases. This case report has been reported in line with the SCARE Guideline [6].

### 1.1 Case presentation

A 56-year-old female presented to the emergency room (ER) with a chief complaint of pain in the right flank area (VAS 4) since one week ago, that worsened one day before hospital admission. The patient underwent DJ stent insertion one year ago and was lost to follow-up, because the patient did not experience complaints with the condition before the pain appeared The patient had hypertension and diabetes mellitus as comorbidities. The patient had a history of previous operations including left nephrectomy, right PCNL, and right right DJ stent placement because of residual stones after right PCNL. Left nephrectomy was performed because the patient's left renal had lost its functional capability.

**Fig. 1:**
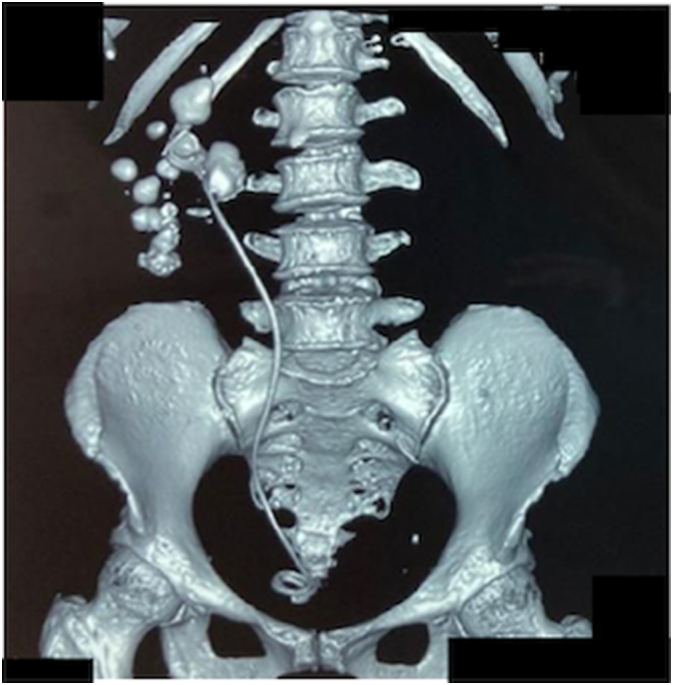
CT stonography 3D reconstruction stonography.

On physical examination, the vital signs were unremarkable. Right renal percussive pain was positive. Pre operative laboratory evaluation showed normal kidney function(1 ng/mL). A non-contrast CT scan revealed hydronephrosis grade 4 and multiple stones in the right kidney with a size of 2.2 × 2.8 × 3 cm (806 HU) and an encrustation of the proximal tip of the right DJ stent (Fig. 1). Complete blood count and renal function were within normal limits. Microscopic stone analysis indicated that the components of the stone were calcium oxalate and uric acid.

**Fig. 2:**
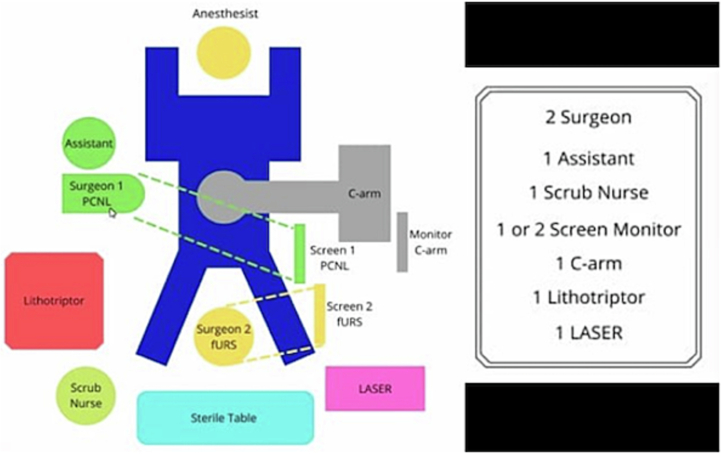
Schematic ECIRS operating room.

**Fig. 3:**
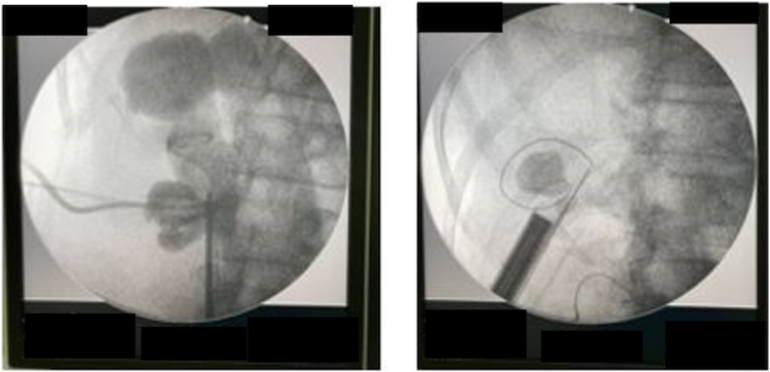
The appearance of kidney stones on fluoroscopy.

**Fig. 4:**
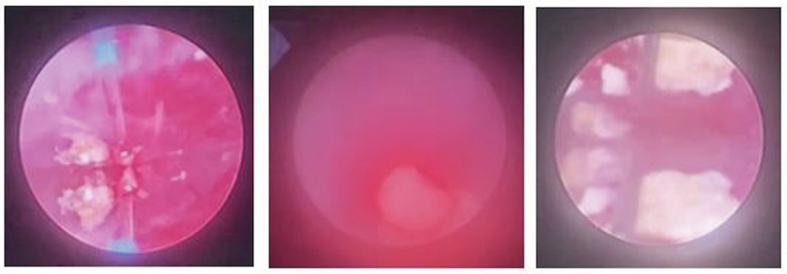
Stone fragmentation using laser lithotripsy.

**Fig. 5:**
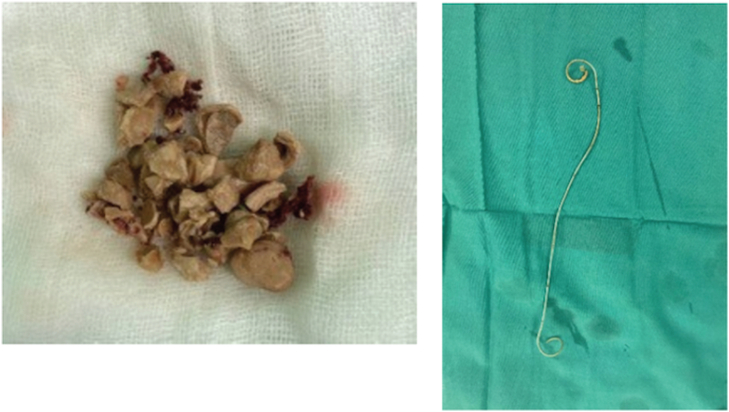
The DJ stent and extracted stone fragments.

**Fig. 6:**
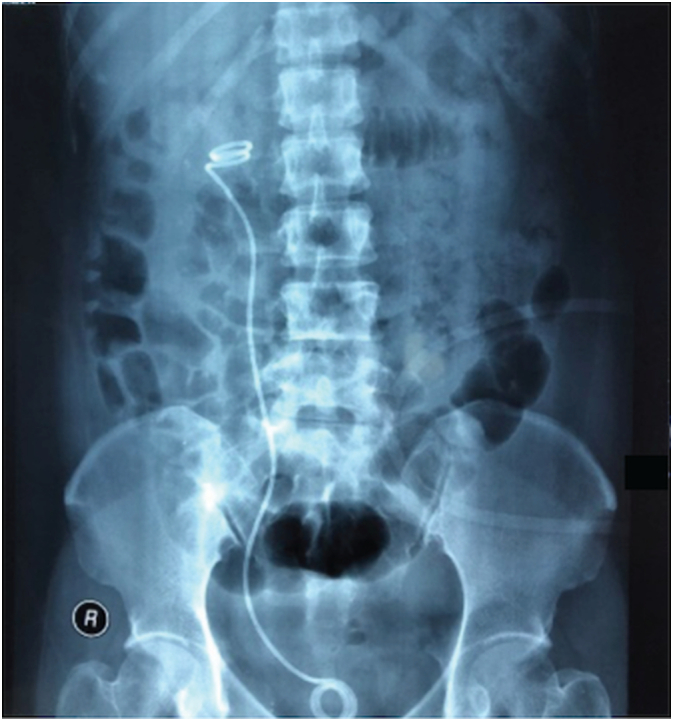
Postoperative x-ray KUB.

The patient then underwent DJ stent removal using the ECIRS technique under general anesthesia. ECIRS was performed by one operator while the other performed URS and removed the DJ stent. An estimated 150 mL of blood were lost during the 45-minute surgery. The patient was set in the supine Valdivia position (Fig. 2). The second operator performed a cystoscopy, obtained a DJ stent image from the right ureter, and then performed a right ureteroscopy to evaluate the right ureter. No stones were found in the right ureter. URS was performed until reaching the right renal pyelum, and there was an encrustation of the proximal tip of the right DJ stent. The procedure was followed by a rigid URS to insert contrast (Urografin), a retrograde pyelogram, and then a puncture to dilate the nephroscope access with C-ARM guiding (dilated fluoroscopy) (Fig. 3). Simultaneously, the first operator performed laser lithotripsy with a Holmium laser (LUMENIS, Germany) (Fig. 4). The stone at the proximal tip was crushed, and the stent was pulled out. The next procedure was the installation of wire, followed by the insertion of the ureteral access sheath with a guiding C-arm and confirmation with fluoroscopy. The flexible ureteroscope was then inserted through the ureteral access sheath, and the stone was visualized. In this procedure, the nephroscope points to the upper pole, while the URS points to the middle pole and pyelum. The ureter was observed in good condition, without any strictures or damage. There is no encrustation on the mid-distal DJ stent (Fig. 5). After removal of the intrarenal stone, the stent was completely removed with a stone grasper. A new DJ stent was inserted into the ureter, and an ECIRS approach was used to ensure the correct positioning of the DJ stent. The surgery completed in 45 min. A follow-up evaluation using a C-arm found no residual stone. After the operation, the patient was discharged from the hospital on the fourth postoperative day with no complications. Two weeks after surgery, DJ stent was removed. The patient was classified as Class 1 by the Clavien-Dindo classification which means that there were mild complications that could be treated without surgery. Urine production showed no abnormalities. Post operative renal function was also normal 0.6 ng/ mL. A x-ray kidney, ureters bladder (KUB) examination after nephrostomy release revealed no residual stone (Fig. 6). One-month follow-up after surgery showed that the right lower back pain had disappeared.

## 2 Discussion

In this report, we present the case of a 56-year-old female with a solitary kidney, a neglected DJ-stent, and multiple recurrent stones using ECIRS. An acquired solitary kidney is found in several conditions, such as after unilateral radical nephrectomy in the adult population, especially in association with living kidney donors, kidney tumors, stones, and trauma [7,8]. In relation to our case, the solitary kidney was acquired due to the previous history of nephrectomy. The nephrectomy was done with the indication of multiple recurrent stones.

Encrustation is a serious problem, especially in the chronically indwelling or neglected stent. DJ stent encrustation was also found in this case. Encrustation of DJ stents can result in irritative voiding symptoms, obstruction of the urinary system, loss of renal function, acute illness, and even death [9]. Patient non-compliance or a lack of follow-up care are the main causes of neglected DJ stents. In order to prevent difficulties, the DJ stent should usually be changed or removed within six weeks to six months [10].

The type of DJ stent that was used in our case was an aliphatic polyurethane-material stent. The combination of findings experienced by this patient consisting of solitary kidney with multiple stones and encrusted DJ stent made this case worthy of being highlighted as a new insight. Previous reports revealed that the type of stent influenced the presence of encrustation. The study reported that 9 % of polyurethane ureteral stents showed signs of encrustation at six weeks, 48 % at 6–12 weeks, and 77 % after 12 weeks [11]. One study reported that a silicone stent is susceptible to the deposition of calcium and magnesium for a period of more than 14 weeks. Bacterial colonization also occurred if the duration of the catheter was longer than 120 days [4].

We performed the ECIRS procedure for the management of our case. ECIRS is a new technique for performing PCNL by combining a simultaneous antero-retrograde approach to the kidney and aiming to treat nephrolithiasis in one step and one access along the urinary tract by utilizing various endourology instruments [5]. ECIRS is a synergistic approach that is a combination of RIRS (retrograde intrarenal surgery) and PCNL. In relation to our case, we performed ECIRS in the case of a neglected DJ stent and recurrent multiple kidney stones in a solitary kidney patient. ECIRS achieves high stone-free rates with low complication rates according to the Clavien–Dindo classification [12]. We reported the safety of ECIRS in our case, in which our patient had only minimal postoperative symptoms and was classified as having only a grade 1 Clavien-Dindo classification. However, there are specific complications associated with ECIRS, including postoperative fever, ileus, wound infection, urinary tract infections (UTIs), stent migration [13,14].

Strategies to prevent neglected DJ stents are crucial in order to reduce and prevent complications. Maintaining a stent register and follow-up system, patients and relatives counselling, showing post-surgery x-ray KUB to patients, active involvement in calling or mailing lost patients, and developing protocols to reduce unnecessary DJ stent placement are all options and strategies to decline the incidence of forgotten DJ stents [15,16].

## 3 Conclusion

The problem of neglected DJ stents is still more prevalent in developing countries, including Indonesia, resulting in significant morbidity and financial burden for patients. The surgical procedures will become more complicated as DJ stents are left in place longer in such cases. ECIRS can be a good interventional option in complex cases like multiple recurrent stones with encrusted DJ-stent in single kidney patients. Providing proper stent registration and good counselling to patients and their families before and after surgical procedure may decline the incidence of neglected DJ stents.

## Additional information

All information is private for this paper.

## Consent

Written informed consent was obtained from the patient’s family for publication of this case report and accompanying images. A copy of the written consent is available for review by the Editor-in-Chief of this journal on request.

## Ethical approval

Ethical approval has been acquired in this study by Health Research Ethics Committee of Dr. Soetomo General-Academic Hospital, Surabaya, Indonesia Ref No : 0505/LOE/301.4.2/VII/2021.

## Funding

This research did not receive any specific grant from funding agencies in the public, commercial, or not-for-profit sectors.

## Author contributions

Yufi Aulia Azmi: Conceptualization, Methodology, Data Curation, Investigation, Writing-Original draft preparation.

Dimas Panca: Conceptualization, Data Curation, Writing-Original draft preparation.

Johan Renaldo: Supervision, Validation, Writing-Reviewing, and Editing.

## Declaration of conflict of interest statement

The authors declare no conflict of interest.

## Guarantor

Johan Renaldo

## Declaration of competing interest

The authors declare that they have no known competing financial interests or personal relationships that could have appeared to influence the work reported in this paper.

## Data Availability

No data was used for the research described in the article.
